# Dichotomic Hippocampal Transcriptome After Glutamatergic vs. GABAergic Deletion of the Cannabinoid CB1 Receptor

**DOI:** 10.3389/fnsyn.2021.660718

**Published:** 2021-04-08

**Authors:** Diego Pascual Cuadrado, Anna Wierczeiko, Charlotte Hewel, Susanne Gerber, Beat Lutz

**Affiliations:** ^1^Institute of Physiological Chemistry, University Medical Center of the Johannes Gutenberg University, Mainz, Germany; ^2^Institute for Human Genetics, University Medical Center of the Johannes Gutenberg University, Mainz, Germany; ^3^Leibniz Institute for Resilience Research (LIR) gGmbH, Mainz, Germany

**Keywords:** CB1 receptor, homeostasis, glutamate transmission, GABA transmission, transcriptome analysis

## Abstract

Brain homeostasis is the dynamic equilibrium whereby physiological parameters are kept actively within a specific range. The homeostatic range is not fixed and may change throughout the individual's lifespan, or may be transiently modified in the presence of severe perturbations. The endocannabinoid system has emerged as a safeguard of homeostasis, e.g., it modulates neurotransmission and protects neurons from prolonged or excessively strong activation. We used genetically engineered mouse lines that lack the cannabinoid type-1 receptor (CB1) either in dorsal telencephalic glutamatergic or in forebrain GABAergic neurons to create new allostatic states, resulting from alterations in the excitatory/inhibitory (E/I) balance. Previous studies with these two mouse lines have shown dichotomic results in the context of behavior, neuronal morphology, and electrophysiology. Thus, we aimed at analyzing the transcriptomic profile of the hippocampal CA region from these mice in the basal condition and after a mild behavioral stimulation (open field). Our results provide insights into the gene networks that compensate chronic E/I imbalances. Among these, there are differentially expressed genes involved in neuronal and synaptic functions, synaptic plasticity, and the regulation of behavior. Interestingly, some of these genes, e.g., *Rab3b, Crhbp*, and *Kcnn2*, and related pathways showed a dichotomic expression, i.e., they are up-regulated in one mutant line and down-regulated in the other one. Subsequent interrogation on the source of the alterations at transcript level were applied using exon-intron split analysis. However, no strong directions toward transcriptional or post-transcriptional regulation comparing both mouse lines were observed. Altogether, the dichotomic gene expression observed and their involved signaling pathways are of interest because they may act as “switches” to modulate the directionality of neural homeostasis, which then is relevant for pathologies, such as stress-related disorders and epilepsy.

## Introduction

Physiological homeostasis is the internal state of a dynamic equilibrium in which an organism functions most optimally. This process includes many parameters being kept within pre-set limits, homeostatic ranges, and the internal mechanisms and feedback loops to respond to internal or external insults (Cooper, 2008). Furthermore, the homeostatic range itself can change under the influence of environmental factors in a process called allostasis, which allows the organism to adapt to the new conditions. Although most of the allostatic changes are transient (e.g., adequate stress response), sometimes different factors establish an entirely new allostatic state, i.e., homeostasis significantly different from most of its conspecifics. The central nervous system (CNS) especially requires tight homeostatic regulation to ensure the correct functioning of neuronal circuits (Woods and Wilson, 2013).

The endocannabinoid system (ECS) acts retrogradely on presynaptic terminals to suppress neurotransmitter release, particularly on-demand after the postsynaptic site's depolarization. Thus, the ECS acts as a feedback loop to maintain homeostasis at the cellular and brain circuit levels. The neuromodulatory action of the ECS is mediated via the activation of the presynaptic cannabinoid receptor-type 1 (CB1), a Gi/o protein-coupled receptor, which activates different intracellular pathways to exert its function. CB1 is expressed in the two major neurotransmitter systems, i.e., in glutamatergic and GABAergic neurons, and many cell types in the brain, including glial cells (Busquets-Garcia et al., 2018; Lutz, 2020).

These observations have fueled numerous studies on the roles of CB1 specifically in different neural populations by using conditional CB1 knock-out (KO) mouse models, where CB1 is inactivated only in distinct neurons (Monory et al., 2006; Lutz et al., 2015; Busquets-Garcia et al., 2016) or glial cells (Han et al., 2012). We have focused mainly on mouse lines that lack CB1 on dorsal telencephalic glutamatergic neurons (Glu-CB1-KO) or forebrain GABAergic neurons (GABA-CB1-KO), which cause an excess of excitation or inhibition, respectively, and forces the brain to adapt to the increased neurotransmission. The use of these mutant mouse lines has shown a differential role for CB1 on glutamatergic and GABAergic neurons, respectively, at different levels, such as THC-induced tetrad effects (Monory et al., 2007; De Giacomo et al., 2020), THC-induced food intake (Bellocchio et al., 2010), THC-induced anxiety-like behavior (Rey et al., 2012), G-protein coupling (Steindel et al., 2013; Mattheus et al., 2016), and long-term hippocampal potentiation (Monory et al., 2015). Interestingly, these studies have also revealed a dichotomic phenotype between Glu-CB1-KO and GABA-CB1-KO animals, which means that each conditional CB1-KO reacts opposingly to the other upon the same stimulus. In some of these studies, it has been observed that CB1-WT and conditional CB1-KO mice showed indistinguishable behavior without prior stimulation (Jacob et al., 2009; Bellocchio et al., 2010; Rey et al., 2012), but only the stimulus revealed the opposing phenotypes. These observations have led us to reason that brain functioning of Glu-CB1-KO and GABA-CB1-KO individuals represent different allostatic states, i.e., homeostasis that is significantly and permanently different from CB1-WT animals. Considering previous research with these mouse lines, we wondered whether the hippocampal transcriptome would show such dichotomic features. To this end, we performed RNA-sequencing of hippocampal Cornus Ammonis (CA) samples from Glu-CB1-KO, GABA-CB1-KO, and corresponding CB1-WT mice. We then conducted a differential expression analysis (DEA) between conditional CB1-KO and CB1-WT mice in the basal state and after a mild behavioral activation. Differentially expressed genes (DEGs) that showed a dichotomic expression when comparing the two mouse lines would be especially relevant, as they could act as “switches” to modulate the directionality of the change in homeostasis, i.e., toward an increased excitatory or inhibitory state, respectively. The discovery of such genes would prove very useful for mental disorders caused due to excessive excitatory or inhibitory drive either in the whole brain or in specific brain regions.

## Materials and Methods

### Mouse Lines

Conditional CB1-KO mice were generated via crossing CB1f/f (JAX Laboratory Stock No. 036107) mice with animals expressing Cre recombinase under the control of the regulatory elements of the genes *dlx5/6* (for deletion in GABAergic terminals; MGI Cat# 3758334, RRID:MGI: 3758334) and *nex1* (for deletion in glutamatergic terminals; MGI Cat# 3758333, RRID:MGI:3758333). This use of the Cre/LoxP system allowed for high specificity in the genetic deletion as shown previously (Monory et al., 2006). Each conditional CB1-KO group was compared to corresponding CB1-WT littermates to minimize possible unwanted variations caused by breeding, handling, age differences, etc. Genotyping was performed as previously described (Massa et al., 2010). Male mice had food and water *ad libitum* during their 12-h light-dark cycle (07:00–19:00) and were 8–10 weeks old when they were sacrificed.

### Behavioral Hippocampal Activation

In order to study how the hippocampus of conditional CB1-KO mice react upon stimulation, we induced neuronal activity during the first half of the light cycle by exposing the animals to an open field arena (40 × 40 × 30 cm white box), a new environment that the mouse could explore undisturbed for 5 min (light intensity: 20–30 lux). This sufficient yet straightforward stimulus-induced neuronal activity throughout the hippocampus during arena exploration induced gene expression changes to be investigated.

### Hippocampal Microdissection and Validation

In order to microdissect only the Cornus Ammonis from the hippocampus, we first removed the dentate gyrus (DG) from the rest of the hippocampus according to the protocol of Hagihara et al. (2009). The remaining hippocampal tissue was then dissected out in one-piece and snap-frozen on dry ice. We selected the CA1/CA2/CA3 region because this is the hippocampal subregion where we reported existing differences in neuronal morphology and electrophysiological properties in these two mutant mouse lines (Monory et al., 2015). Moreover, we attempted to reduce the intrinsic heterogeneity observed within the hippocampus by excluding the DG due to distinct functionalities, e.g., in neurogenesis and as a gate input from the entorhinal cortex.

### RNA Extraction and Reverse Transcription

In order to sustain the amount and quality of the hippocampal RNA for sequencing, the extraction and purification of RNA was performed as previously described (Lerner et al., 2018). To summarize, we followed the instructions from the RNeasy Mini kit (Qiagen) with a slight modification during the homogenization step. Frozen tissue was homogenized in 600 μL RLT buffer with 1% β-mercaptoethanol (according to the manufacturer's protocol), though with the addition of 200 μL chloroform. Apart from this step, there were no other changes made. Obtained RNA samples were treated with DNase I to degrade any possible contamination by genomic DNA. Samples were then eluted in 30 μL RNase-free water. The working bench and the tools used were cleaned before and during the extraction with RNase away plus (MßP, San Diego, CA, USA) to avoid any RNA degradation.

To perform qPCR, 1 μg of RNA was retrotranscribed into complementary DNA (cDNA) with the high-capacity cDNA reverse transcription kit (Life Technologies, Germany). This kit uses random primer hexamers for the reverse-transcription step. The resulting cDNA was diluted 1:10 in RNase-free water and stored at −80°C.

### Hippocampal CA1/CA2/CA3 RNA-Sequencing

The hippocampal transcriptome sequencing occurred at the Core Facility Genomics (Institute of Molecular Biology gGmbH, IMB; Mainz). Next generation sequencing (NGS) library preparation was performed in two steps. First, cDNA was generated using NuGEN's Ovation RNA-seq system v2, from an input amount of 10 ng of total RNA, following the kit's instructions from the year 2012 (NuGEN, The Netherlands). Samples were amplified using the single primer isothermal amplification (SPIA) method from NuGEN. The resulting purified cDNA was quantified using the Qubit dsDNA HS assay kit in a Qubit 2.0 Fluorometer (Life Technologies, Germany). Afterward, the cDNA was profiled on a high sensitivity DNA chip using a 2100 Bioanalyzer (Agilent Technologies, Germany) as quality control. From the total cDNA, 1.5 μg were fragmented using a Covaris S2 focused-ultrasonicator (Covaris, UK), with the following parameters: (1) Duty cycle = 10%; (2) Intensity = 5; (3) Cycles/Burst = 200; (4) Time = 160 s; and (5) Water level = 15. After the fragmentation, the resulting material was once again quantified and profiled using a Qubit 2.0 and a 2100 Bioanalyzer, respectively, as described above. This process was repeated as an extra quality control point to ensure an optimal fragmentation.

Secondly, NGS libraries were generated from 100 ng of fragmented cDNA using NuGEN's Ovation ultralow system v2, following the kit's manual from the year 2014 (NuGEN, The Netherlands). Libraries were amplified in 7 PCR cycles and purified using beads. These purified libraries were quantified as described above, and profiled on a DNA 1000 Chip using a 2100 Bioanalyzer as the last quality control. Libraries representing all the experimental groups were pooled into individual pools containing 12 libraries, all of them in equimolar ratio. Each pool was loaded into four lanes of an Illumina's HiSeq flowcell and ran on a HiSeq 2500 in High-output mode (Illumina, USA), generating single-reads 50 base pairs long with an average yield of 52 million reads per library.

### Quantitative Polymerase Chain Reaction (qPCR)

For the qPCR procedure, cDNA was amplified using the commercial TaqMan assays (Applied Biosystems) with an ABI7300 real-time PCR cycler (Applied Biosystems). Reactions were performed in duplicates, and β*-actin* was used as a reference gene. Analysis of the resulting data was performed using the 7300 system SDS software (Applied Biosystems).

Genes and their respective primers were selected according to the necessities of the experiment. *Arc* was used to measure hippocampal activation, whereas *TDO2* and *Lphn2* were used to prove the tissue purity resulting from the hippocampal microdissection. The rest of the genes (except for the reference gene) were chosen because they appeared as differentially expressed genes in the RNA-seq analysis (Table 1). We used an independent batch of mice (*n* = 7–9 animals per group) to validate genes of interest from our RNA-seq data.

**Table 1:** List of TaqMan primers used for qPCR.

**Gene symbol**	**Gene name**	**TaqMan primer code**
β-actin	β-Actin	Mm00607939_s1
Arc	Activity-regulated cytoskeleton-associated protein	Mm01204954_g1
TDO2	Tryptophan 2,3-dioxygenase	Mm00451266_m1
Lphn2	Latrophilin 2	Mm01320597_m1
Npy	Neuropeptide Y	Mm00445771_m1
CRHBP	Corticotropin-releasing hormone binding protein	Mm01283832_m1
Cnr1	Cannabinoid receptor type-1	Mm01212171_s1
FosB	FBK osteosarcoma oncogene B	Mm00500401
Bdnf (exon V)	Brain-derived neurotrophic factor	Mm04230607
Nr4a2	Nuclear receptor subfamily 4, group A, member 2	Mm00443060_m1
Grin2B	Glutamate receptor ionotropic NMDA-type subunit 2B	Mm00433820_m1

*For each gene, the pair of primers was selected according to the manufacturer's suggestion and their gene coverage*.

### Statistical Analysis of Behavioral and qPCR Data

Continuous variables were graphically represented as individual values and mean ± standard error of the mean (SEM). First, normality was checked for each parameter analyzed in this study. Next, conditional CB1-KO groups were statistically compared to their respective CB1-WT littermates with an unpaired *t*-Test (including Welch's correction when variances were significantly different) or a non-parametric Mann-Whitney test, depending on whether samples were normally distributed or not, respectively. Statistical analysis was performed with GraphPad Prism v5.0 (RRID:SCR_002798) and InVivoStat v4.1. *P*-values were two-tailed, and differences were considered statistically significant if the *p*-value was below 0.05.

### Bioinformatic Analysis

An initial quality check of the raw sequencing data was done via FastQC version (v0.11.8) (RRID:SCR_014583). Then, bbduk.sh from the BBMap (BBmap, RRID:SCR_016965) suite of tools (version 38.06) was employed to perform adapter trimming and quality filtering of the raw sequence reads (q30 cutoff) (Zerbino et al., 2018). Afterward, the trimmed and cleaned sequences were mapped against the mouse reference genome mm10 from UCSC (downloaded via Illumina iGenome: http://support.illumina.com/sequencing/sequencing_software/igenome.html, download date 06/06/18), using the STAR aligner (version 2.6.0a) (RRID:SCR_015899) with default options (Dobin et al., 2013). Summarized quality results were created by MultiQC (version 1.8.dev0) (Ewels et al., 2016). Counting of reads per gene was done via featureCounts (version 1.6.2), with the -s 2 option, using the annotation file for the mm10 mouse genome from iGenomes (Liao et al., 2014).

The R package DESeq2 (version 1.30.0) was used for all subsequent differential gene expression (DGE) (Love et al., 2014). *P*-values were adjusted using the the Benhamini-Hochberg method for multiple testing correction (Benjamini and Hochberg, 1995), and the threshold for significantly differentially expressed genes (DEGs) was defined as the adjusted *p*-value (p-adjusted) < 5 %.

The subsequent Gene Ontology (GO) term analysis was executed by the R package clusterProfiler (version 3.18.0) using the standard over-representation test (Yu et al., 2012). All GO terms with an adjusted *p* < 5 % were considered significantly overrepresented for the respective gene subset.

In order to check the cell specificity of the DEGs resulting from the Glu-CB1 and GABA-CB1 comparisons, we downloaded the transcriptomic information of different brain cell types of *Mus musculus*, derived from single-cell RNA-seq analyses and published by the Allen Institute for Brain Sciences (https://portal.brain-map.org/atlases-and-data/rnaseq). The cell type specific expression patterns of the respective genes were extracted and visualized using the R package pheatmap (version 1.0.12) (Supplementary Figures 1–4).

EISA (exon-intron split analysis) by Gaidatzis et al. (2015) was performed using the EisaR package with standard settings, as per the vignette (Gaidatzis et al., 2015). The same significance threshold for post-transcriptionally regulated genes was used as previously described for the DGE analysis. Finally, the Pearson correlation between the log_2_ Fold changes derived from exons and introns was calculated per gene.

All plots were realized with the R packages ggplot2 (version 3.3.2) or venn (version 1.9) (Wickham, 2016; Dusa, 2020).

## Results

### Open Field Paradigm to Induce Neuronal Activity and Sample Selection for RNA-Sequencing

**Figure 1:**
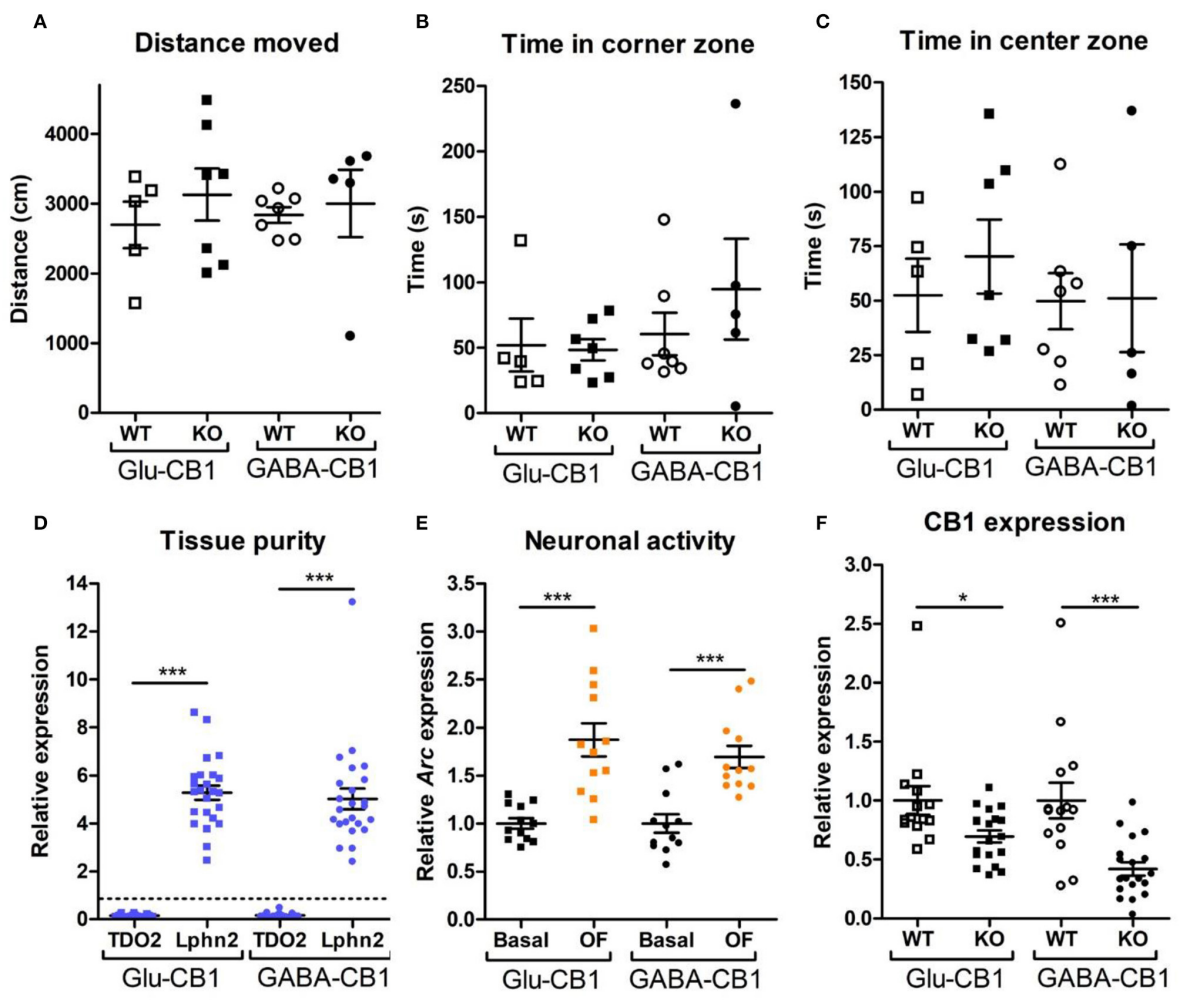
Behavioral and qPCR data used for the selection of the final samples. Samples for RNA-sequencing were selected depending on the purity of the dissected tissue, as well as the anxiety-like behavior and the neuronal activity induced by the exposure to the open field. All animals that are shown were selected for the transcriptomic analysis. **(A)** Locomotion activity in the open field arena (statistical power = 9.06%; calculated by one-way ANOVA power analysis). **(B)** Time spent in the corner zones (anxiety-like behavior) of the open field arena (statistical power = 7.92%; calculated by one-way ANOVA power analysis). **(C)** Time spent in the center zone (anxiolytic-like behavior) of the open field arena (statistical power = 9.81%; calculated by one-way ANOVA power analysis). **(D)** Gene expression levels for the gene activity-regulated cytoskeleton-associated protein (*Arc*) showed increased neuronal activity upon brief exposure to a new environment (open field arena). CB1-WT and their respective conditional CB1-KO samples were pooled together in each of the groups shown. **(E)** The purity of the tissue resulting from the microdissection of the hippocampus was checked by measuring the expression levels of *tryptophan 2,3-deoxygenase* (*TDO2*) and *latrophilin 2* (*Lphn2*), which are specific for the DG and the CA. The graph shows only CA tissue samples that were normalized and compared to the respective DG samples (dashed line). **(F)** Gene expression levels of *CB1* (*cnr1*) in the CA regions of CB1-WT mice and the respective conditional CB1-KO animals. Samples from basal state and open field group were pooled together. Bars represent mean ± S.E.M. Unpaired *t*-Test with Welch's correction when variances between groups were different. *p < 0.01; ***p < 0.001.

For the transcriptomic analysis, we used 8–10 week-old male mice at basal (home cage) conditions with the following genotype and group size: Glu-CB1-WT (*n* = 5), Glu-CB1-KO (*n* = 7), GABA-CB1-WT (*n* = 6), GABA-CB1-KO (*n* = 6). For the open field stimulated state, mice were behaviorally characterized (Figures 1A–C). The group size of the open field groups were: Glu-CB1-WT (*n* = 5), Glu-CB1-KO (*n* = 7), GABA-CB1-WT (*n* = 5), and GABA-CB1-KO (*n* = 7). No significant genotype and treatment differences were observed in the 5-min open field exposure, neither in general locomotion (Figure 1A) nor in anxiety-like behavior (Figures 1B,C). Isolated hippocampi of all mice used for transcriptomic analysis were checked for the purity of the CA1/CA2/CA3 region by using RT-qPCR for *latrophilin 2* (*Lphn2*), a marker for the CA region. In contrast, *tryptophan 2,3-deoxygenase* (*TDO2*), a marker for dentate gyrus, was not detected (Figure 1D). Furthermore, the open field stimulus was monitored by observing significant increases in *Arc* mRNA levels (Figure 1E). CB1 deletion in mutants was substantiated by observing decreased *cnr1 (CB1)* mRNA levels (Figure 1F).

### Dichotomies in the Transcriptomic Profiles of Glu-CB1-KO and GABA-CB1-KO Hippocampi in the Basal State

First, we analyzed the transcriptome differences between the allostatic states, represented by Glu-CB1-KO and GABA-CB1-KO hippocampi, and their respective homeostatic states, as represented by CB1-WT littermates at basal, i.e., unstimulated home cage conditions.

**Figure 2:**
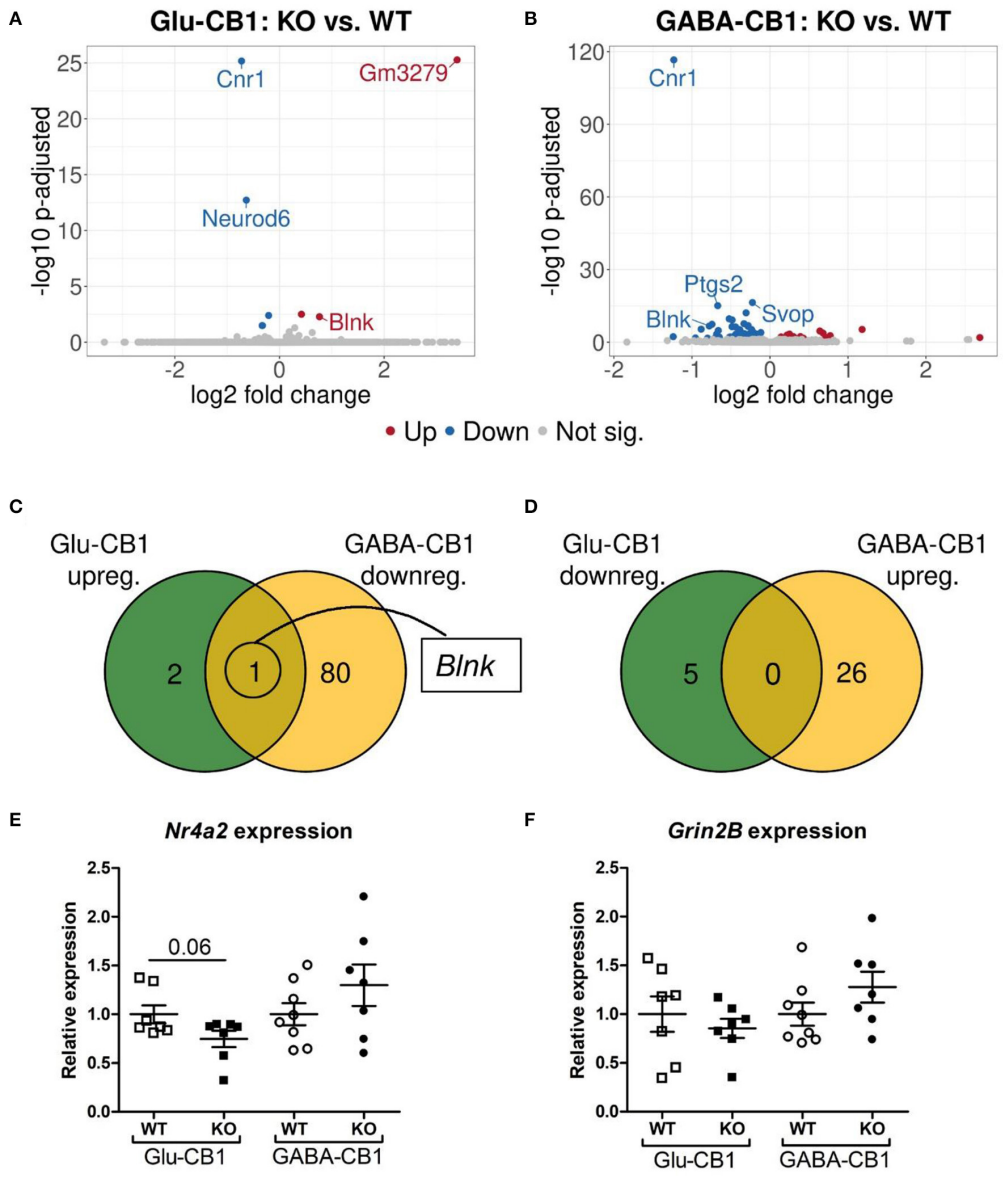
Transcriptomic profiles from Glu-CB1-KO and GABA-CB1-KO mice in the basal state. **(A,B)** Volcano plots showing differentially expressed genes (red and blue dots) found when comparing Glu-CB1-KO **(A)** and GABA-CB1-KO **(B)** mice to their respective CB1-WT in their basal state. Red dots represent upregulated genes resulting from this comparison, whereas blue dots indicate downregulated genes. Labels of the top3, dichotomic, and qPCR-validated DEGs are shown. Non-significant genes are colored in gray. **(C,D)** Venn diagrams to visualize the number of dichotomic genes found between Glu-CB1 and GABA-CB1 DEGs in the basal state, i.e., those genes that appear up-regulated in one of the conditional CB1-KO lines and down-regulated in the other, or vice versa. **(E)** Expression levels derived from qPCR of the transcription factor *Nr4a2* for each of the genotypes analyzed in the basal state. **(F)** Expression levels derived from qPCR of the gene *Grin2B* for each of the genotypes analyzed in the basal state. RT-qPCR data normalized to β*-actin*. Bars represent mean ± SEM. Unpaired t-Test with Welch's correction when variances between groups were different.

The differential expression analysis (DEA) revealed several differentially expressed genes (DEGs) when comparing the conditional CB1-KO mice with their respective CB1-WT littermates. The relatively low level of differences in expression, as evident by the number of differentially regulated genes above the significance threshold and the PCA plots of the test conditions, is in accordance with previous observations that conditional CB1-KO mice are very similar to wild-type animals in the absence of a stimulus (Jacob et al., 2009; Rey et al., 2012). Interestingly, we observed a strong difference in the number of DEGs between the mutant lines. While, we found 8 DEGs in the Glu-CB1 mouse line (Figure 2A), we observed 107 DEGs in the GABA-CB1 comparison (Figure 2B). Thus, we found only one overlapping dichotomous gene between the different analysis groups, namely the gene *B-cell linker* (*Blnk*) (Figures 2C,D). We further analyzed genes related to synaptic function and plasticity via RT-qPCR. Here, *Nr4a2*, a critical transcription factor for neuronal function, and *Grin2B*, a subunit of the glutamate NMDA receptor, were investigated as DEGs in an independent set of RNA samples (Figures 2E,F). Interestingly, no genes related to the ECS were found to be differentially expressed when comparing each conditional CB1-KO group to their respective CB1-WT controls.

To find out whether the DEGs from Glu-CB1 and GABA-CB1 comparisons are exclusively expressed within certain cell-types, we extracted the expression patterns of the respective genes from the transcriptomic information of mouse brain-cell types published by the Allen Institute for Brain Sciences (https://portal.brain-map.org/atlases-and-data/rnaseq). We could not observe common patterns of gene expression in the DEGs resulting from the Glu-CB1 comparison (Supplementary Figure 1), most likely due to the low number of genes. However, the *in-silico* analysis revealed that most of the DEGs found in the GABA-CB1 comparison are expressed in both glutamatergic and GABAergic neurons. However, a small proportion of DEGs from this comparison are expressed mainly in glutamatergic populations (Supplementary Figure 2). We next performed a gene ontology (GO) enrichment analysis with the DEGs found. This analysis provided an insight into which biological processes, cell compartments, and molecular functions are altered, possibly also in compensation of the loss of CB1 in these two neuronal populations by increasing either excitatory or inhibitory neurotransmission, present in Glu-CB1-KO and GABA-CB1-KO hippocampi, respectively. Due to the low number of DEGs found when comparing Glu-CB1-KO and CB1-WT hippocampi in the basal state (Figure 2A), we could not allocate any overrepresented processes in this dataset. However, we found overrepresented GO terms of downregulated genes in GABA-CB1-KO mice compared with respective CB1-WT controls (Table 2). These processes comprised the terms of synaptic transmission, as well as learning and memory. Moreover, GO terms related to neuronal structures, such as neuronal projections, axonal structures, and secretory vesicles, were also found to be significantly enriched for downregulated genes in GABA-CB1-KO as compared to CB1-WT. In conclusion, our results showed significant differences between GABA-CB1-KO and Glu-CB1-KO animals in their basal state, presumably in adapting to increased inhibitory and excitatory neurotransmission, respectively.

**Table 2:** GO terms of the DEGs from GABA-CB1-KO mice in the basal state.

	**GABA-CB1-KO in the basal state**	**Enrichment**	***P*-value (adjusted)**
Biological process	Modulation of chemical synaptic transmission	Down-regulated	0.04375221
	Regulation of trans-synaptic signaling	Down-regulated	0.04375221
	Learning or memory	Down-regulated	0.04375221
	Memory	Down-regulated	0.04375221
Cell compartment	Secretory vesicle	Down-regulated	0.02787115
	Axon terminus	Down-regulated	0.02787115
	Axon part	Down-regulated	0.02787115
	Neuron projection terminus	Down-regulated	0.02787115
	Distal axon	Down-regulated	0.02787115

*Table showing the list of GO terms resulting after analyzing the DEGs in the comparison GABA-CB1-KO vs. CB1-WT in the basal state. GO terms marked as downregulated indicate that these terms are enriched for downregulated DEGs, and the opposite holds true for terms marked as upregulated*.

### Exposure to Open Field Induces Different Transcriptional Profiles in Glu-CB1-KO and GABA-CB1-KO Mice

**Figure 3:**
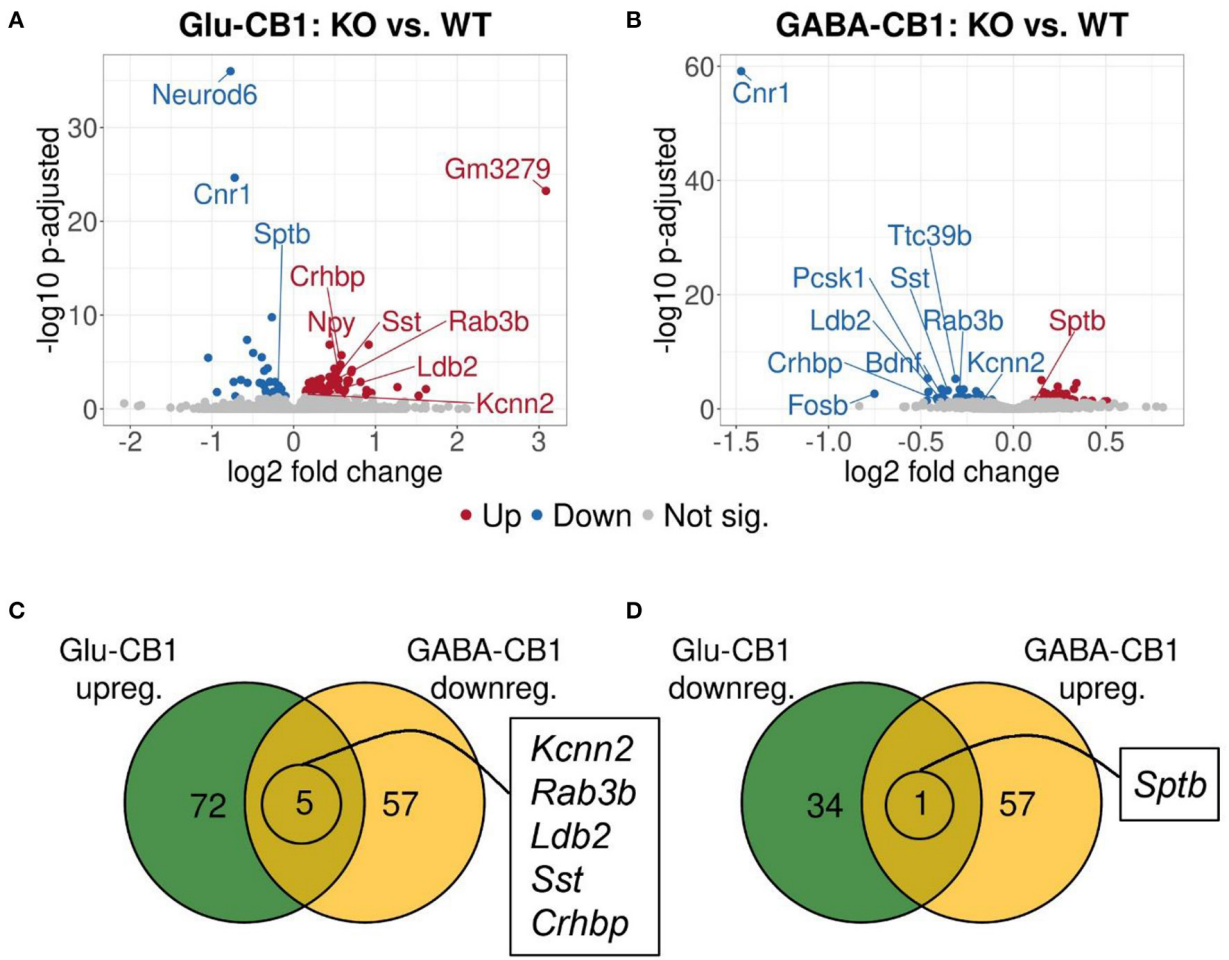
Transcriptomic profiles from Glu-CB1-KO and GABA-CB1-KO mice after open field exposure. **(A,B)** Volcano plots showing differentially expressed genes (red and blue dots) found when comparing Glu-CB1-KO **(A)** and GABA-CB1-KO **(B)** mice to their respective CB1-WT after exposure to the open field arena. Red dots represent upregulated genes resulting from these comparisons, whereas blue dots indicate downregulated genes. Labels of the top3, dichotomic, and qPCR-validated DEGs are shown. Non-significant genes are colored in gray. **(C,D)** Venn diagrams to visualize the number of dichotomic genes found between Glu-CB1-KO and GABA-CB1-KO data after open field exposure, i.e., those genes that appear up-regulated in one of the conditional CB1-KO lines and down-regulated in the other, or vice versa.

**Figure 4:**
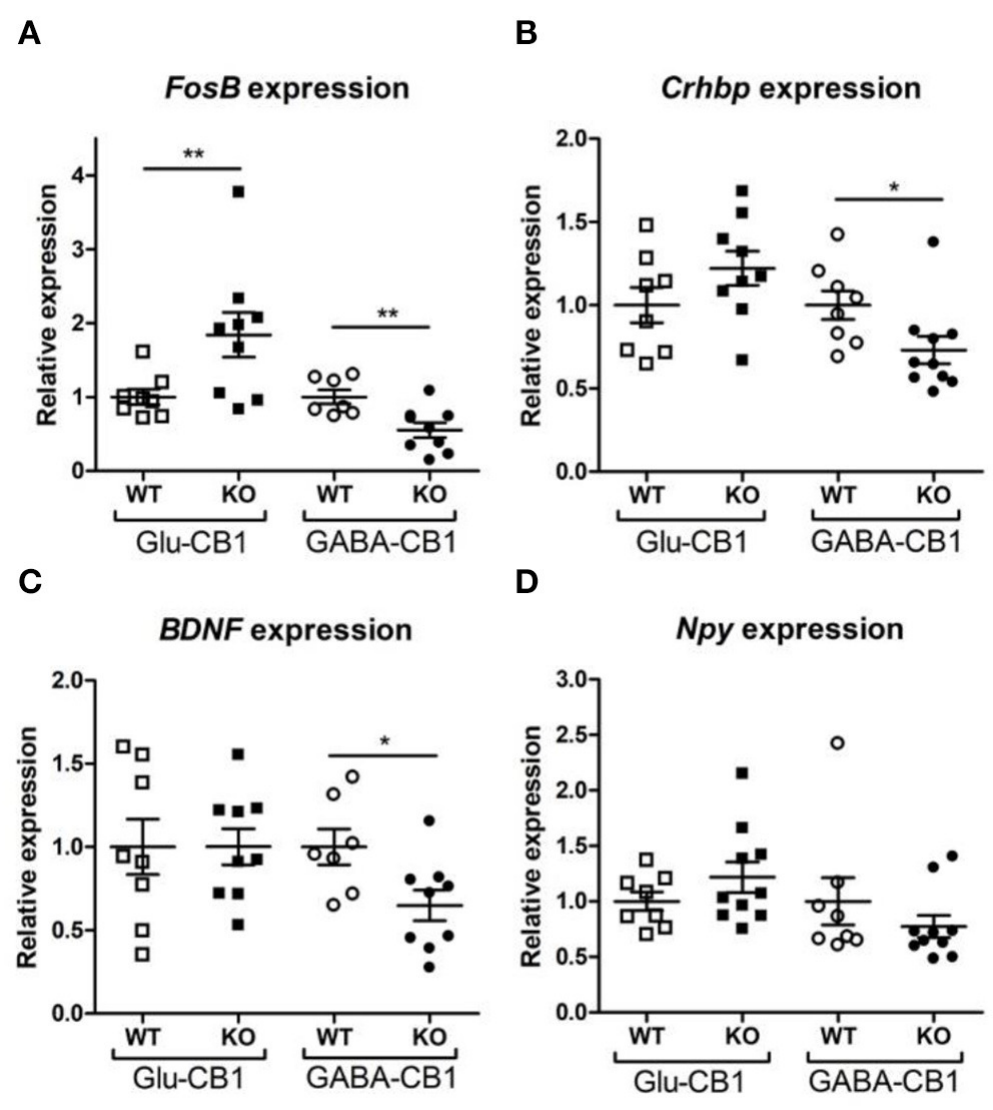
Relative expression of genes of interest after open field exposure measured via RT-qPCR. The measurements were performed with independent samples (*n* = 7–9 replicates per group) to validate the results from our RNA-seq experiment. Genes that showed a potential dichotomy between the comparisons of both mouse lines were selected for further validation. **(A)** Expression levels of *FosB* proto-oncogene, AP-1 transcription factor subunit. **(B)** Expression levels of *corticotropin-releasing hormone binding protein* (Crhbp). **(C)** Expression levels of *brain-derived neurotrophic factor* (*BDNF*). **(D)** *Expression levels of neuropeptide Y* (*Npy*). The expression levels were normalized using β*-actin* as housekeeping gene. Bars represent mean ± S.E.M. Unpaired *t*-Test with Welch's correction when variances between groups were different. *p < 0.05; **p < 0.01.

A brief exposure to a new environment (e.g., open field arena) triggers neuronal activity in the hippocampus (Cohen et al., 2017). In order to study how each of the allostatic and homeostatic states, respectively, react upon such a stimulation, we exposed both Glu-CB1 and GABA-CB1 mice for 5 min to an open field arena (Figure 3) and isolated the tissue 60 min afterwards for transcriptomic analysis. The subsequent DEA revealed more DEGs in this stimulated condition than in the basal state. Thus, the open field exposure is sufficient to induce gene expression changes, although transcriptional differences between conditional CB1-KO and CB1-WT samples were relatively low. Interestingly, we found more genes that show dichotomic expression after open field exposure (Figures 3C,D) than in the basal state (Figures 2C,D), in particular for the Glu-CB1-KO mice. This observation suggests activity-induced neuronal pathways being modulated in the hippocampi of both Glu-CB1-KO and GABA-CB1-KO mice, although in opposing directions. Among the dichotomically regulated genes, we found transcription factors related to neuronal developmental processes (e.g., *Ldb2*), potassium channels involved in Ca^2+^ homeostasis (e.g., *Kcnn2*), neuropeptides (e.g., *Sst*), and proteins involved in the biogenesis of neuronal vesicles (e.g., *Rab3b*), the regulation of the cytoskeleton (e.g., *Sptb*) and the regulation of the stress response (e.g., C*rhbp*). In addition, we investigated via RT-qPCR (Figures 4A–D) the dichotomic expression levels of additional genes after behavioral activation, such as *FosB* (Figure 4A), *Crhbp* (Figure 4B), *BDNF* (Figure 4C), and *Npy* (Figure 4D).

The cell-type specificity analysis revealed that the DEGs resulting from the Glu-CB1 comparison were expressed in different neuronal populations. Interestingly, some of these DEGs had very high levels of expression in GABAergic neurons, suggesting that the lack of CB1 in Glu-CB1-KO mice caused some form of dysregulation on GABAergic populations (Supplementary Figure 3). In the case of the GABA-CB1 comparison, almost all DEGs were expressed in both glutamatergic and GABAergic populations (Supplementary Figure 4). Next, we analyzed GO term overrepresentations within those DEGs found after exposure to the open field arena. Contrary to their basal state, Glu-CB1-KO mice provided interesting GO terms after behavioral activation of the hippocampus (Table 3). The upregulated genes from the Glu-CB1 comparison were revealed to be significantly involved in the activity of adrenergic and G-protein coupled receptors, as well as neuropeptide hormones. The signaling pathways of the glutamate receptor and neuropeptides were also enriched for those upregulated genes, as well as different neuronal structures such as the dendritic shaft, dopaminergic synapses, the axon, and secretory granules. Interestingly, those genes that revealed to be downregulated comparing Glu-CB1-KO and CB1-WT mice after open field exposure were involved in voltage-gated potassium channel complexes. Lastly, biological processes such as the fear response, axon guidance or the regulation of neurotransmitter transport were enriched for upregulated genes observed in the comparison of Glu-CB1-KO with CB1-WT hippocampi after a mild stimulus.

**Table 3:** GO terms of the DEGs from Glu-CB1-KO mice after open field.

	**Glu-CB1-KO after open field**	**Enrichment**	***P*-value (adjusted)**
Molecular function	Receptor regulator activity	Up-regulated	0.0028834
	Receptor ligand activity	Up-regulated	0.00453663
	Neuropeptide hormone activity	Up-regulated	0.00453663
	G protein-coupled amine receptor activity	Up-regulated	0.01502056
	Adrenergic receptor activity	Up-regulated	0.03844831
Biological process	Glutamate receptor signaling pathway	Up-regulated	0.0285555
	Multicellular organismal response to stress	Up-regulated	0.0285555
	Axon guidance	Up-regulated	0.0285555
	Fear response	Up-regulated	0.03192236
	Regulation of neurotransmitter transport	Up-regulated	0.03192236
	Neuropeptide signaling pathway	Up-regulated	0.03192236
Cell compartment	Perikaryon	Up-regulated	0.00049563
	Axon terminus	Up-regulated	0.00841979
	Dendritic shaft	Up-regulated	0.00841979
	Axon part	Up-regulated	0.02405893
	Secretory granule	Up-regulated	0.03819382
	Dopaminergic synapse	Up-regulated	0.04162235
	Voltage-gated potassium channel complex	Down-regulated	0.04554603
	Potassium channel complex	Down-regulated	0.04554603

*Table showing the list of GO terms resulting after analyzing the DEGs in the comparison Glu-CB1-KO vs. CB1-WT after open field exposure. GO terms marked as downregulated indicate that these terms are enriched for downregulated DEGs, and the opposite holds true for terms marked as upregulated*.

In contrary, for DEGs in GABA-CB1-KO mice, only downregulated genes yielded enriched terms (Table 4). These terms included processes for a proper neuronal function, such as GABAergic and dopaminergic synaptic transmission, as well as the regulation of trans-synaptic signaling, the membrane potential, and the action potential. Specific neuronal structures (e.g., synapses and axons) were also found to be overrepresented for the downregulated genes in our analysis.

**Table 4:** GO terms of the DEGs from GABA-CB1-KO mice after open field.

	**GABA-CB1-KO after open field**	**Enrichment**	***P*-value (adjusted)**
Biological process	Synaptic transmission, GABAergic	Down-regulated	0.00588666
	Regulation of synaptic transmission, GABAergic	Down-regulated	0.0133165
	Regulation of action potential	Down-regulated	0.02260484
	Modulation of chemical synaptic transmission	Down-regulated	0.02260484
	Regulation of trans-synaptic signaling	Down-regulated	0.02260484
	Synaptic transmission, dopaminergic	Down-regulated	0.02260484
	Action potential	Down-regulated	0.02260484
	Regulation of membrane potential	Down-regulated	0.03951345
	Behavior	Down-regulated	0.0467256
Cell compartment	Presynapse	Down-regulated	0.00600701
	Distal axon	Down-regulated	0.01692391
	Secretory granule	Down-regulated	0.01719666
	Integral component of synaptic membrane	Down-regulated	0.01719666
	Secretory vesicle	Down-regulated	0.01719666
	Neuron projection terminus	Down-regulated	0.02740205
	GABA-ergic synapse	Down-regulated	0.02756174
	Axon part	Down-regulated	0.01719666

*Table showing the list of GO terms resulting after analyzing the DEGs in the comparison GABA-CB1-KO vs. CB1-WT after open field exposure. GO terms marked as downregulated indicate that these terms are enriched for downregulated DEGs, and the opposite holds true for terms marked as upregulated*.

### Interrogation on the Source of the Transcriptional Alterations Applying Exon-Intron Split Analysis (EISA)

**Figure 5:**
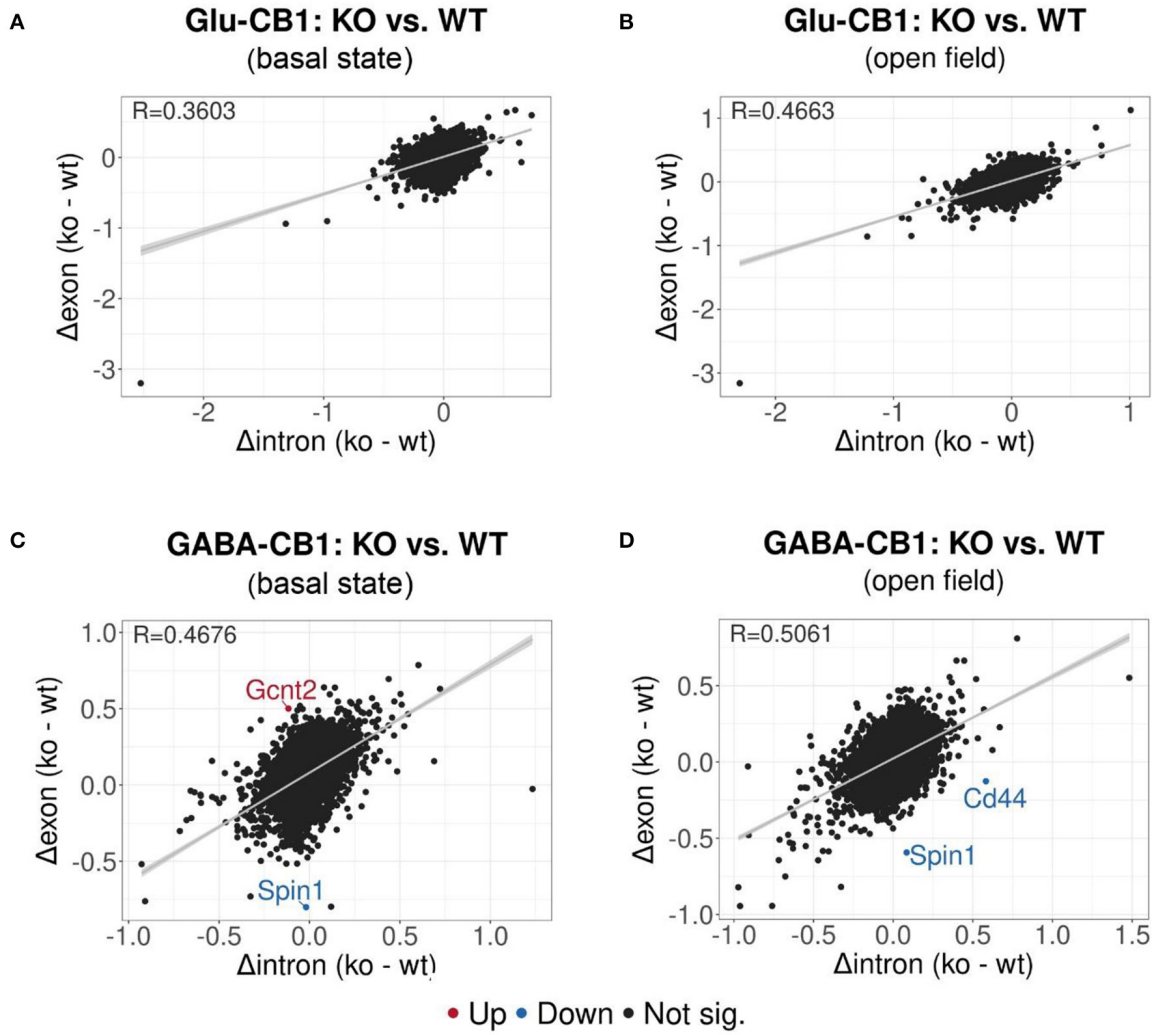
EISA analysis of the Glu-CB1 and GABA-CB1 comparisons in the basal state and after open field exposure. Dot plots representing the change in exonic and intronic counts for each gene (dots). The value R at the top left corner of each graph indicates the slope of the regression curve, as well as the tendency on whether the overall changes in gene expression are due to transcriptional (R = 1) or posttranscriptional (R = 0) changes. **(A)** EISA data resulting from comparing Glu-CB1-KO vs. CB1-WT mice in the basal state. **(B)** EISA data resulting from comparing Glu-CB1-KO vs. CB1-WT mice after open field exposure. **(C)** EISA data resulting from comparing GABA-CB1-KO vs. CB1-WT mice in the basal state. **(D)** EISA data resulting from comparing GABA-CB1-KO and CB1-WT mice after open field exposure. Significant post-transcriptionally regulated genes comparing conditional CB1-KO and CB1-WT mice are labeled and colored in red for upregulation and blue for downregulation.

EISA was conducted to determine whether the gene expression changes are due to transcriptional or post-transcriptional mechanisms via the analysis of intronic and exonic read changes in each comparison (Figure 5) (Gaidatzis et al., 2015). The Pearson correlation between intronic and exonic changes indicates whether the global gene expression differences originated at the transcriptional or posttranscriptional level, as represented by the correlation coefficient (R). A normal cell relies on a mixture of transcriptional or posttranscriptional mechanisms to regulate its gene expression. We performed EISA on each conditional CB1-KO mouse line compared to their WT littermates and found that only a few genes were found to be significantly post-transcriptionally regulated (Figure 5). Thus, we cannot pinpoint the overall origin of the transcriptomic changes observed either to dominantly transcriptional or post-transcriptional control. However, it is interesting to note that the Glu-CB1 line (Figures 5A,B) had slightly lower R-values than the GABA-CB1 line. This observation could indicate increased post-transcriptional regulation in Glu-CB1-KO mice to counterbalance the excess of excitatory neurotransmission. Nevertheless, more experiments are required to confirm this point.

## Discussion

Brain homeostasis and the modulation of neurotransmission is tightly connected to the ECS (Busquets-Garcia et al., 2018; Lutz, 2020). This function of the ECS, mediated by the CB1 receptor, is fundamental both at the neuronal and the circuit level, in order to avoid neuronal overexcitation and maladaptive behaviors (Lau and Tymianski, 2010; Olloquequi et al., 2018). Consequently, the ECS is used as a therapeutic target for many pathologies, and thus, to explore the mechanisms by which the ECS acts. However, the main receptor of the ECS (CB1) has a ubiquitous expression in the mammalian brain, which complicates the study of cell-specific mechanisms underlying CB1 action. Conditional CB1-KO mouse lines, in which CB1 is deleted in a specific neuronal or glial population, have boosted our knowledge of the ECS in specific cell populations. Thus, we used these mouse lines to study brain homeostasis and potential gene expression changes whereby the brain of these mice tries to counteract the imbalances of excitatory and inhibitory neurotransmission, as represented by Glu-CB1-KO and GABA-CB1-KO mice, respectively.

Interestingly, at basal state (home cage condition), we could observe over a hundred DEGs in GABA-CB1-KO hippocampi when comparing them to their respective CB1-WT animals, whereas we found only eight DEGs in Glu-CB1-KO samples. This observation suggests important differences in the adaptive mechanisms used by GABA-CB1-KO, rather directed toward promoting neuronal and network excitability, and by Glu-CB1-KO animals, rather directed toward compensating increased excitability and excitotoxicity. Thus, the differences in the number of DEGs indicate different compensatory strategies, some of which depend more than others on transcriptional processes. Moreover, the mere number of DEGs may not reflect the functional importance of the actual DEGs in the homeostatic response. Furthermore, we found that some genes also had a dichotomic expression when comparing Glu-CB1-KO and GABA-CB1-KO data, such as *Nr4a2*, an important transcription factor in neurons, and *Grin2B*, a subunit of the NMDA glutamate receptor. *Nr4a2* and *Grin2B* are especially relevant, as they are heavily involved in synaptic transmission and signaling (Endele et al., 2010; Rajan et al., 2020). *Grin2B* has a central role on long-term potentiation and synaptic plasticity (Keith et al., 2019), network excitability (Marquardt et al., 2019), and can promote neuronal health or regulate excitotoxicity (Hardingham and Bading, 2010). GO terms related to synaptic transmission and signaling, and the neuronal structures relevant for cell-cell communication were enriched for downregulated genes in GABA-CB1-KO samples compared to their CB1-WT counterparts. However, even at basal state, the transcriptome differences between mutants and wild-types were very mild, though, at the morphological level, e.g., dendritic branching, are strongly different (Monory et al., 2015), indicating that the accumulation of life events and environmental challenges are involved in these morphological alterations. The lack of expression changes in ECS-related genes could result from adaptive mechanisms during mouse development in order to secure the functioning of the ECS in the basal state, as they also lack CB1 during this life period. Moreover, our behavioral stimulus was brief and mild. It could be that more intense or long-lasting stimuli could induce wider changes in the transcriptomic profile that would ultimately affect the expression levels of ECS-related genes.

Along this line, we aimed at studying how these conditional CB1-KO mice react upon a mild challenge, represented by a brief exposure to an open field arena. This new environment was sufficient to induce changes in the neuronal gene expression patterns in Glu-CB1-KO and GABA-CB1-KO mice, as neuronal activity triggered an excess of excitatory or inhibitory neurotransmission, respectively. We found over a hundred DEGs in both comparisons and, surprisingly, several more genes showed a dichotomic expression between mouse lines compared to the basal state. We did not find any commonly upregulated or downregulated genes between comparisons, except for CB1. This suggests that no common mechanism was used by Glu-CB1-KO and GABA-CB1-KO mice to try restoring homeostasis. Among the dichotomic genes, we found genes involved in neuropeptide signaling (*Crhbp, Npy, Sst*), transcription factors (*Ldb2, FosB*), the cytoskeleton (*Sptb*), synaptic function (*Rab3b, Kcnn2*), or synaptic plasticity (*Bdnf* ). *Sst*, e.g., is used as a neurotransmitter by inhibitory interneurons and is essential for the correct maturation of inhibitory synapses (Su et al., 2020) and for behavioral control (Anaclet et al., 2018). The neuropeptide Y (*Npy*) is also present in inhibitory interneurons throughout the brain and regulates behavioral responses, especially those related to anxiety and fear (Desai et al., 2014; Bartsch et al., 2020). Both *Sst* and *Npy* modulate the activity of neural networks through their modulation of GABAergic interneurons. *Rab3b* is also an important regulator of excitability, as it is known to prime synaptic vesicles release (Schlüter et al., 2006) and is required for endocannabinoid-dependent long-term depression at inhibitory synapses (Tsetsenis et al., 2011). Lastly, the potassium channel *Kcnn2* is critical for neuronal Ca^2+^ dynamics and homeostasis (Richter et al., 2016), thus modulating brain and neuronal processes such as synaptic plasticity (Sun et al., 2020). The relevance of these genes for neuronal cells means that these genes, or others along their respective pathways, could serve to modulate brain homeostasis towards an increased excitatory or inhibitory state or back to homeostasis.

Our *in-silico* cell-type specificity analysis showed that most of these DEGs were similarly expressed across all brain cell types. However, a proportion of DEGs found in the GABA-CB1 comparison in the basal state were only expressed in glutamatergic neurons. Interestingly, we observed a similar feature when analyzing the DEGs from the Glu-CB1 comparison after open field, as some of these DEGs had very high levels of expression in GABAergic populations. These results suggest that the lack of CB1 on a specific neuronal population not only alters the transcriptomic profile of said population, but also has an important effect on the transcriptomic landscape of other neuronal cell types. Another interesting observation is that the GO enrichment analysis performed on the DEGs found after open field exposure yielded very different results for each comparison. On the one hand, those GO terms among the upregulated genes in the Glu-CB1 suggest an increase in the activity and signaling of various receptors and neuropeptides, and the regulation of neurotransmitter transport. The upregulation of these genes could be a consequence of neuronal activity in an excessively excitatory environment. Voltage-gated potassium channel complex was the only GO term to be enriched for downregulated genes in this comparison. On the other hand, those genes that were downregulated in the GABA-CB1 comparison were significantly enriched for processes related to synaptic transmission and action potential, as well as the regulation of trans-synaptic signaling and the membrane potential. The downregulation of these genes could be the direct result of increased inhibitory drive induced by neuronal activity and the absence of CB1 on GABAergic neurons.

In summary, our results showed differences between the transcriptomic profiles of Glu-CB1-KO and GABA-CB1-KO mice in their basal state and after exposure to a mild stimulus. Furthermore, we found several genes with dichotomic expression levels when comparing both mouse lines (i.e., they are increased in one mouse line and decreased in the other). Understanding how these genes or their respective pathways are regulated and their interactions could shed new light into how the brain maintains its homeostasis, as well as into neuronal mechanisms protecting against E/I imbalances.

## Data Availability Statement

The original contributions presented in the study are publicly available. This data can be found in NCBI under the GEO accession number GSE168873 (https://www.ncbi.nlm.nih.gov/geo/query/acc.cgi?acc=GSE168873).

## Ethics Statement

The animal study was reviewed and approved by Landesuntersuchungsamt Rheinland-Pfalz.

## Author Contributions

DP performed all behavioral and molecular experiments, except the sequencing of RNA. AW and CH performed all bioinformatic analysis of the sequencing data. DP wrote the manuscript with the support of AW and BL. All authors contributed to the article and approved the submitted version.

## Conflict of Interest

The authors declare that the research was conducted in the absence of any commercial or financial relationships that could be construed as a potential conflict of interest.
