# Supplementary figures and images for: Dichotomic Hippocampal Transcriptome After Glutamatergic vs. GABAergic Deletion of the Cannabinoid CB1 Receptor
Source: Front Synaptic Neurosci. 2021 Apr 8;13:660718. doi: 10.3389/fnsyn.2021.660718 (PMC8060565; doi:10.3389/fnsyn.2021.660718)

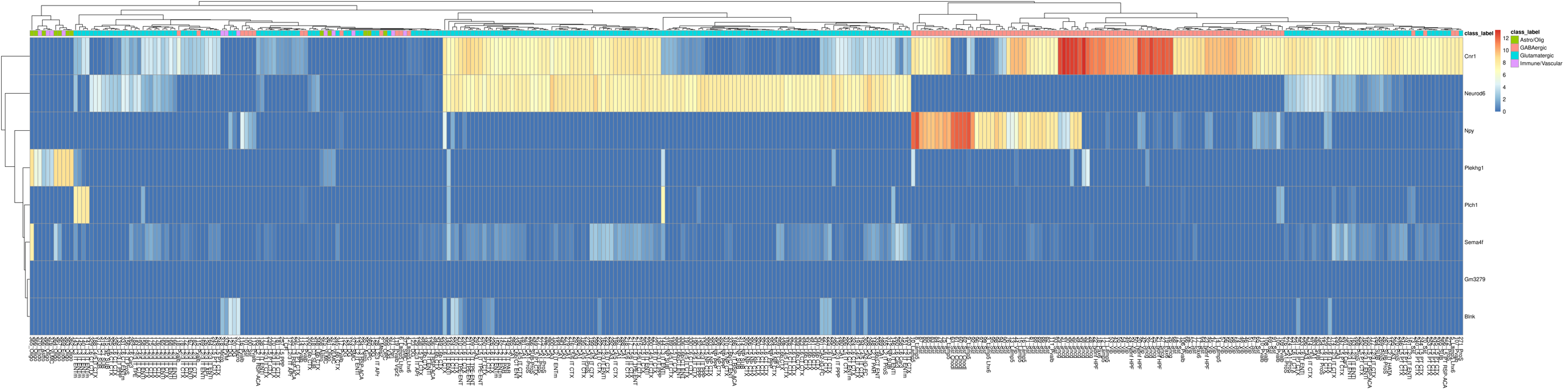

Supplement: Supplementary file 1 [file Image_1.PNG]
