# Supplementary material for: Dichotomic Hippocampal Transcriptome After Glutamatergic vs. GABAergic Deletion of the Cannabinoid CB1 Receptor
Source: Front Synaptic Neurosci. 2021 Apr 8;13:660718. doi: 10.3389/fnsyn.2021.660718 (PMC8060565; doi:10.3389/fnsyn.2021.660718)
Supplement: Supplementary file 5 [file Data_Sheet_1.PDF]

Supplementary Figure 1: *In-silico* analysis of cell type specificity for DEGs found after comparing Glu-CB1-KO with their respective CB1-WT in the basal state. We used the transcriptomic information derived from single-cell RNA-seq analyses published by the Allen Institute for Brain Sciences (https://portal.brain-map.org/atlases-and-data/rnaseq). The heatmap represents the expression levels of the DEGs found in our RNA-seq analysis across different glutamatergic and GABAergic populations, as well as non-neuronal cell-types. The expression levels are color coded, with blue indicating low expression and red being indicative for high expression.

Supplementary Figure 2: *In-silico* analysis of cell type specificity for DEGs found after comparing GABA-CB1-KO with their respective CB1-WT in the basal state. We used the transcriptomic information derived from single-cell RNA-seq analyses published by the Allen Institute for Brain Sciences (https://portal.brain-map.org/atlases-and-data/rnaseq). The heatmap represents the expression levels of the DEGs found in our RNA-seq analysis across different glutamatergic and GABAergic populations, as well as non-neuronal cell-types. The expression levels are color coded, with blue indicating low expression and red being indicative for high expression.

Supplementary Figure 3: *In-silico* analysis of cell type specificity for DEGs found after comparing Glu-CB1-KO with their respective CB1-WT after open field exposure. We used the transcriptomic information derived from single-cell RNA-seq analysespublished by the Allen Institute for Brain Sciences (https://portal.brain-map.org/atlases-and-data/rnaseq). The heatmap represents the expression levels of the DEGs found in our RNA-seq analysis across different glutamatergic and GABAergic populations, as well as non-neuronal cell-types. The expression levels are color coded, with blue indicating low expression and red being indicative for high expression.

Supplementary Figure 4: *In-silico* analysis of cell type specificity for DEGs found after comparing GABA-CB1-KO with their respective CB1-WT after open field exposure. We used the transcriptomic information derived from single-cell RNA-seq analyses published by the Allen Institute for Brain Sciences (https://portal.brain-map.org/atlases-and-data/rnaseq). The heatmap represents the expression levels of the DEGs found in our RNA-seq analysis across different glutamatergic and GABAergic populations, as well as non-neuronal cell-types. The expression levels are color coded, with blue indicating low expression and red being indicative for high expression.
